# Enhanced Cycling Stability through Erbium Doping of LiMn_2_O_4_ Cathode Material Synthesized by Sol-Gel Technique

**DOI:** 10.3390/ma11091558

**Published:** 2018-08-29

**Authors:** Hongyuan Zhao, Xiuzhi Bai, Jing Wang, Dongdong Li, Bo Li, Yashuang Wang, Li Dong, Binbin Liu, Sridhar Komarneni

**Affiliations:** 1 School of Mechanical & Electrical Engineering, Henan Institute of Science and Technology, Xinxiang 453003, China; 2 Research Branch of Advanced Materials & Green Energy, Henan Institute of Science and Technology, Xinxiang 453003, China; Lidongdong1994@126.com (D.L.); boli9277@163.com (B.L.); yashuangwang1102@126.com (Y.W.); ledong181255@163.com (L.D.); LiuBinbin4118@163.com (B.L.); 3 School of Chemistry and Chemical Engineering, Henan Institute of Science and Technology, Xinxiang 453003, China; amibai@126.com; 4 School of Chemical and Material Engineering, Jiangnan University, Wuxi 214122, China; jingwang@jiangnan.edu.cn; 5 Materials Research Institute and Department of Ecosystem Science and Management, 204 Energy and the Environment Laboratory, Pennsylvania State University, University Park, PA 16802, USA

**Keywords:** cathode material, LiMn_2_O_4_, Er-doping, sol-gel method, cycling stability

## Abstract

In this work, LiMn_2−x_Er_x_O_4_ (x ≤ 0.05) samples were obtained by sol-gel processing with erbium nitrate as the erbium source. XRD measurements showed that the Er-doping had no substantial impact on the crystalline structure of the sample. The optimal LiMn_1.97_Er_0.03_O_4_ sample exhibited an intrinsic spinel structure and a narrow particle size distribution. The introduction of Er^3+^ ions reduced the content of Mn^3+^ ions, which seemed to efficiently suppress the Jahn–Teller distortion. Moreover, the decreased lattice parameters suggested that a more stable spinel structure was obtained, because the Er^3+^ ions in a ErO_6_ octahedra have stronger bonding energy (615 kJ/mol) than that of the Mn^3+^ ions in a MnO_6_ octahedra (402 kJ/mol). The present results suggest that the excellent cycling life of the optimal LiMn_1.97_Er_0.03_O_4_ sample is because of the inhibition of the Jahn-Teller distortion and the improvement of the structural stability. When cycled at 0.5 C, the optimal LiMn_1.97_Er_0.03_O_4_ sample exhibited a high initial capacity of 130.2 mAh g^−1^ with an excellent retention of 95.2% after 100 cycles. More significantly, this sample showed 83.1 mAh g^−1^ at 10 C, while the undoped sample showed a much lower capacity. Additionally, when cycled at 55 °C, a satisfactory retention of 91.4% could be achieved at 0.5 C after 100 cycles with a first reversible capacity of 130.1 mAh g^−1^.

## 1. Introduction

With increasing environmental awareness, many people have realized the importance of green travel, which is very useful for reducing environmental pollution and protecting human health. As an optimal choice for green travel, electric vehicles with rechargeable batteries have become very popular all over the world. Meanwhile, lithium-ion batteries, as the power source, have been developed quickly in recent years [1,2,3,4,5,6,7,8]. It is generally known that there are four major classes of mature cathode materials, namely LiCoO_2_ [9,10], LiFePO_4_ [11,12], LiNi_1−x−y_Co_x_M_y_O_2_ (M = Mn, Al) [13,14], and LiMn_2_O_4_ [15,16], for batteries. Among these materials, LiMn_2_O_4_ shows many virtues such as mature production technology, cheap production costs, non-pollution characteristics, and so forth [17,18,19,20]. However, the large-scale commercial applications of this material have been seriously restricted because of its poor cycling life and high-temperature performance, which are mostly a consequence of Jahn–Teller distortion, manganese dissolution, and non-uniform particle-size distribution [7,21,22,23,24]. Therefore, there is a tremendous need to optimize this material for better performance.

Among the existing, numerous solutions, many researchers generally prefer surface modification and cation doping [17,25,26,27,28,29,30]. The surface modification can make a positive contribution to the improvement of the cycling life by sealing off the active material from the electrolyte corrosion to suppress the manganese dissolution. However, this strategy cannot fundamentally inhibit the Jahn–Teller distortion [25,26]. Thus, the optimization effect of surface modification is limited. Therefore, many researchers have chosen to use the cation doping strategy to optimize the cycling life of LiMn_2_O_4_ [31,32,33]. Yu et al. [34] prepared the Li_1+x_Mn_2−x_O_4_ samples by a solid-state sintering method. The obtained Li_1.06_Mn_1.94_O_4_ sample with extra Li showed better cycling performance, because the introduction of lithium ions can weaken the ordering of lithium ions and enhance the structural stability of the sample. Huang et al. [31] reported the synthesis of LiCu_x_Mn_2−x_O_4_ by a low-temperature molten-salt combustion method, and their results showed that the Cu-doping can optimize the average particle size and size distribution. More importantly, the Cu substitution significantly improved the cycling performance. Furthermore, the LiAl_x_Mn_2−x_O_4_ samples synthesized by a solution combustion technique showed better cycling life as a result of the reduced Jahn-Teller distortion by Al-doping [35]. These results indicated that the cycling life of LiMn_2_O_4_ could be improved by doping with other cations. Such optimization effects have been corroborated well by others [32,36,37]. In addition, the synthetic method could have a significant influence on the electrochemical properties. So far, the LiMn_2_O_4_ cathode materials have been obtained by a solid-state method [38,39], hydrothermal method [40,41], combustion method [31,42,43], co-precipitation method [44], sol-gel method [45,46,47], and so on. Among these methods, the sol-gel method is highly suitable to prepare the high-performance, doped LiMn_2_O_4_ due to the following advantages: (1) the reactants are evenly mixed at the molecular level in the process of forming a gel; (2) uniform cation doping can be achieved due to the intimate mixing of chemical contents in a solution; and (3) the chemical reaction is carried out easily, because the diffusion of the components is on a nanometer scale, which requires relatively low synthetic temperature.

In this work, the LiMn_2−x_Er_x_O_4_ (x ≤ 0.05) samples were successfully synthesized by the sol-gel technique with erbium nitrate as the erbium source. The influence of the erbium-doping content on the structures, morphologies, and electrochemical properties of the LiMn_2−x_Er_x_O_4_ samples obtained by the sol-gel process is discussed. The optimal Er-doped LiMn_2_O_4_ sample showed the intrinsic spinel structure and narrow particle size distribution. More importantly, this sample exhibited excellent cycling stability, superior high-rate capability, and outstanding high-temperature performance.

## 2. Materials and Methods

**Figure 1:**
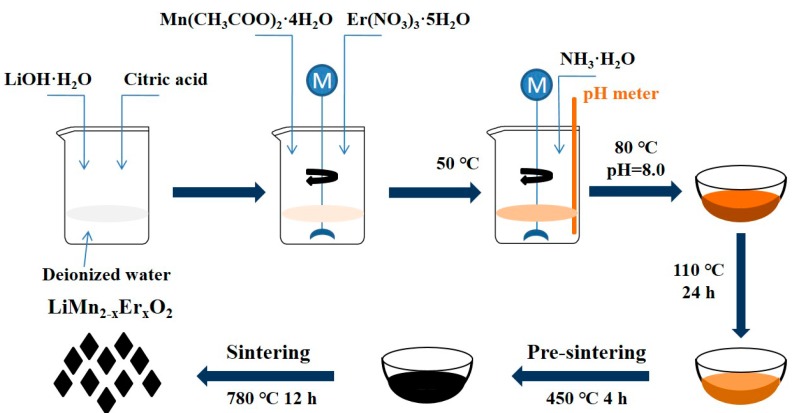
Schematic illustration of the synthesis of the LiMn_2−x_Er_x_O_4_ (x = 0, 0.01, 0.03, 0.05) samples.

The LiMn_2−x_Er_x_O_4_ samples were obtained by a sol-gel process with erbium nitrate (Sinopharm Chemical Reagent Co., Ltd., Shanghai, China) as the erbium source. Figure 1 shows the schematic illustration of the synthesis of these Er-doped LiMn_2_O_4_ samples. Firstly, stoichiometric lithium hydroxide (Sinopharm Chemical Reagent Co., Ltd., Shanghai, China) and citric acid (Sinopharm Chemical Reagent Co., Ltd., Shanghai, China) were weighed to prepare the mixed solution. Under vigorous stirring, a mixed solution of erbium nitrate and manganese acetate (Sinopharm Chemical Reagent Co., Ltd., Shanghai, China) was added dropwise into the abovementioned solution at 50 °C. After continuous stirring for 30 min, NH_3_·H_2_O (Sinopharm Chemical Reagent Co., Ltd., Shanghai, China) was added dropwise into the mixed solution to adjust the pH to 8.0, and the temperature was subsequently adjusted to 80 °C. After further stirring for a few hours, a reddish-brown sol was formed, which was then dried at 110 °C. The obtained dried gel was calcined at 450 °C for 4 h and then further heated at 780 °C for 12 h.

The crystal structures of the obtained erbium-doped spinels were studied by X-ray diffraction (XRD, Bruker DX-1000, Karlsruhe, Germany) with Cu Kα radiation (λ = 0.15406 nm). The lattice parameters of these erbium-doped samples were obtained by using MDI Jade 5.0 software. The surface morphologies and microstructures were determined by using scanning electron microscopy (SEM, JEOL JSM-6360LV, Tokyo, Japan) with an energy dispersive X-ray spectrometer (EDX, EDAX Inc., Mahwah, NJ, USA). X-ray photoelectron spectroscopy (XPS) was obtained by using a Thermo ESCALAB 250XI instrument (Thermo Fisher Scientific, Waltham, MA, USA) with a monochromatic Al Ka (1486.6 eV) X-ray source.

The active electrode consisted of the obtained erbium-doped spinels, conductive acetylene black, and polyvinylidene fluoride (weight ratio = 85:10:5). The anode material and diaphragm were lithium foil and Celgard 2400 polymer (Celgard, Charlotte, NA, USA), respectively. A mixture of 1 M of LiPF_6_, ethyl methyl carbonate (EMC), dimethyl carbonate (DMC), and ethylene carbonate (EC) was used as the electrolyte (V_EMC_:V_DMC_:V_EC_ = 1:1:1, (Guangzhou Tinci Materials Technology Co., Ltd., Guagnzhou, China)). The electrochemical measurements were carried out on a NEWARE battery testing system (NEWARE, Shenzhen, China). The electrochemical impedance spectroscopy (EIS) was carried out by using a CS-350 electrochemical workstation (Wuhan Corrtest Instruments Crop., Ltd., Wuhan, China). The impedance plots were recorded by applying an AC (alternating current) voltage of 5 mV amplitude in the frequency range of 0.1–100 kHz.

## 3. Results and Discussion

**Figure 2:**
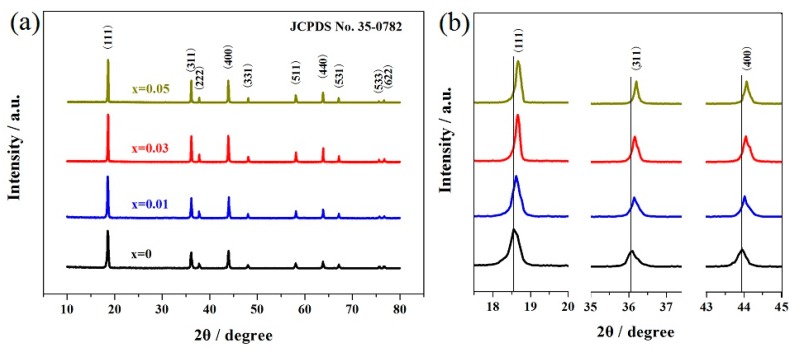
XRD results of the LiMn_2−x_Er_x_O_4_ (x = 0, 0.01, 0.03, 0.05) samples: (**a**) the consecutive XRD patterns and (**b**) the representative magnified XRD patterns.

Figure 2 presents the XRD results of the LiMn_2−x_Er_x_O_4_ (x = 0, 0.01, 0.03, 0.05) samples obtained by the sol-gel method. As shown in Figure 2a, all the Er-doped LiMn_2_O_4_ samples showed the obvious characteristic diffraction peaks of spinel-type lithium manganese oxide (JCPDS No. 35-0782), suggesting that the introduction of a small amount of erbium ions did not have detectable influence on the material’s structure [35,42]. All the LiMn_2−x_Er_x_O_4_ (x = 0.01, 0.03, 0.05) samples maintained the inherent spinel structure of LiMn_2_O_4_. According to the previously reported results [21,45] and according to the reported references [21,48], the (220) peak of LiMn_2_O_4_ is particularly sensitive to the other cations at tetrahedral sites (8a). If the doped ions inhabit the tetrahedral sites, the (220) peak should appear in the corresponding XRD pattern. However, the (220) peak cannot be observed in the XRD patterns in all the LiMn_2−x_Er_x_O_4_ (x = 0, 0.01, 0.03, 0.05) samples. This indicates that the erbium ions replaced the manganese ions at the octahedral sites in the Er-doped LiMn_2_O_4_ samples.

Table 1 lists the corresponding crystal parameters of these samples. The lattice parameters of these erbium-doped samples were obtained by using MDI Jade 5.0 software. It is obvious from these data that all the Er-doped LiMn_2_O_4_ samples possessed a Fd-3m space group. As the Er-doping content increased, the LiMn_2−x_Er_x_O_4_ (x = 0.01, 0.03, 0.05) samples showed smaller lattice parameters and cell volumes. Figure 2b shows the magnified map of the (111), (311), and (400) peaks. It can be clearly seen that the introduction of erbium ions caused a shift toward the higher angle, which further indicated the decrease of the crystal parameters. These results suggest the formation of a more stable spinel structure [20,49]. This is principally because the Er^3+^ ions in the ErO_6_ octahedra showed stronger bonding energy (615 kJ/mol) than that of the Mn^3+^ ions in the MnO_6_ octahedra (402 kJ/mol) [50]. In addition, it should be noted that the Er-doped LiMn_2_O_4_ samples showed higher (311)/(400) peak intensity ratios, which have much to do with the cycling life of LiMn_2_O_4_ [21,51]. An analysis of the previously published results indicated that the introduction of erbium ions may play a constructive role in enhancing the electrochemical properties.

**Figure 3:**
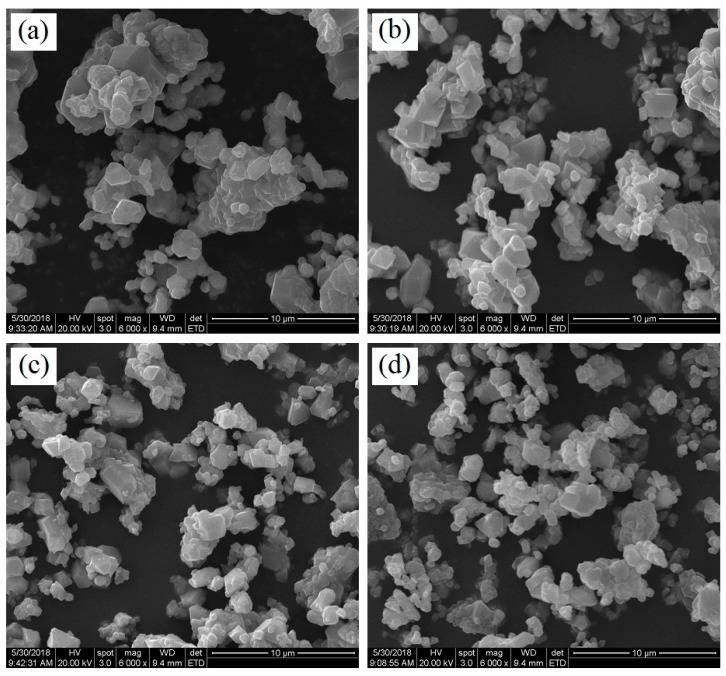
SEM images of the LiMn_2−x_Er_x_O_4_ samples: (**a**) x = 0; (**b**) x = 0.01; (**c**) x = 0.03; and (**d**) x = 0.05.

**Figure 4:**
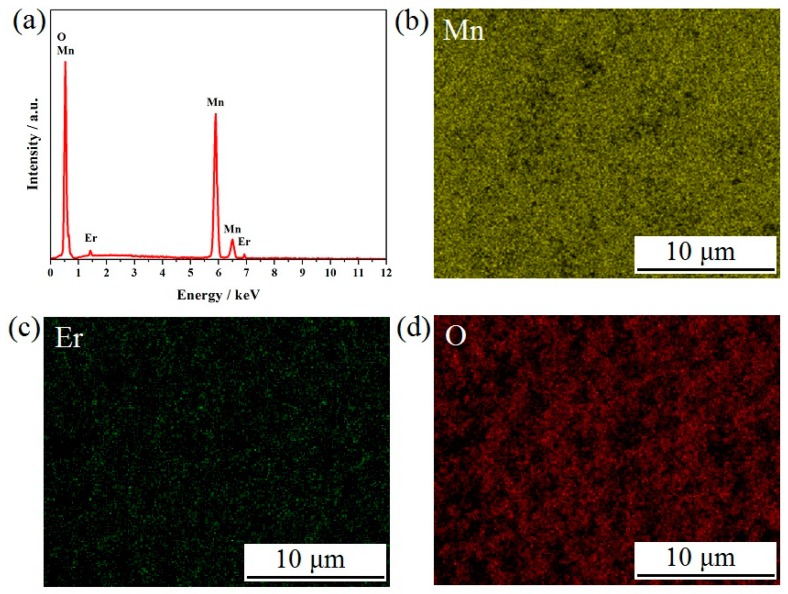
(**a**) SEM-EDX pattern and (**b**–**d**) SEM-mapping results of the LiMn_1.97_Er_0.03_O_4_ sample.

Figure 3 presents the SEM images of the LiMn_2−x_Er_x_O_4_ (x = 0, 0.01, 0.03, 0.05) samples obtained by the sol-gel technology. It can be clearly observed that the introduction of the erbium ions had a certain influence on the surface morphology of the powders. For the undoped LiMn_2_O_4_ particles shown in Figure 3a, the particle size distribution was unsatisfactory because of severe particle agglomeration. By contrast, all the Er-doped LiMn_2_O_4_ particles (Figure 3b–d) showed relatively good surface morphology with relatively little particle agglomeration. When the Er-doping content increased, the mean diameter of the LiMn_2−x_Er_x_O_4_ (x = 0.01, 0.03, 0.05) samples showed a decreasing tendency. In particular, the LiMn_1.97_Er_0.03_O_4_ particles shown in Figure 3c presented the most uniform size distribution, which is conducive to the enhancement of cycling life [21,28,32]. These results indicate that the introduction of erbium ions can effectively optimize the size distribution, which contributes to the improvement of the cycling stability. Figure 4 shows the SEM-EDX pattern and SEM-mapping results of the LiMn_1.97_Er_0.03_O_4_ sample as a representative sample of the Er-doped LiMn_2_O_4_ samples. The SEM-EDX pattern shown in Figure 4a can confirm the successful incorporation of erbium ions in the doped LiMn_2_O_4_ samples. As shown in Figure 4b–d, the SEM-mapping results show the homogeneous distribution of the manganese, erbium, and oxygen elements in the Er-doped LiMn_2_O_4_ samples.

**Figure 5:**
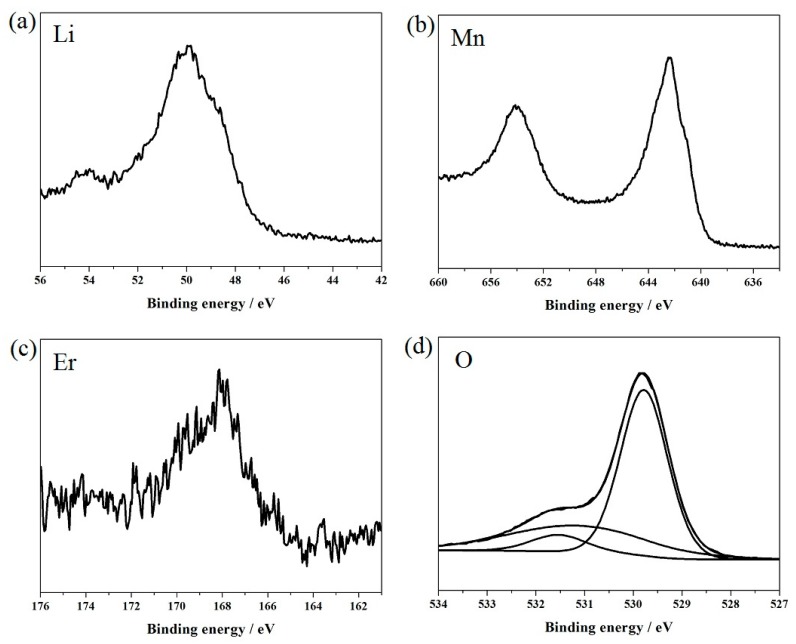
XPS spectra of Li1s, Mn2p, Er4d, and O1s in the LiMn_1.97_Er_0.03_O_4_ sample.

Figure 5 shows the XPS spectra of Li1s, Mn2p, Er4d, and O1s in the LiMn_1.97_Er_0.03_O_4_ sample, which was selected as a representative sample of the Er-doped LiMn_2_O_4_ samples. The binding energy peaks of the Li1s, Mn2p, and O1s are well shown in Figure 5a,b,d and coincide with the previous reported literature [21]. It is important to note that the Mn2p_3/2_ binding energy of the manganese element was at 642.4 eV. However, according to the existing literature [36,52], the Mn2p_3/2_ binding energies of the trivalent and tetravalent manganese ions are at 641.7 eV and 643.1 eV, respectively. Thus, it can be inferred that the manganese element in the LiMn_1.97_Er_0.03_O_4_ sample corresponded to the coexistence state of the trivalent and tetravalent manganese ions. As for the erbium element, the binding energy peak shown in Figure 5c corresponded to the oxidation states for Er4d, which was assigned to Er^3+^ at 168.8 eV, which agrees with the previous result [53].

**Figure 6:**
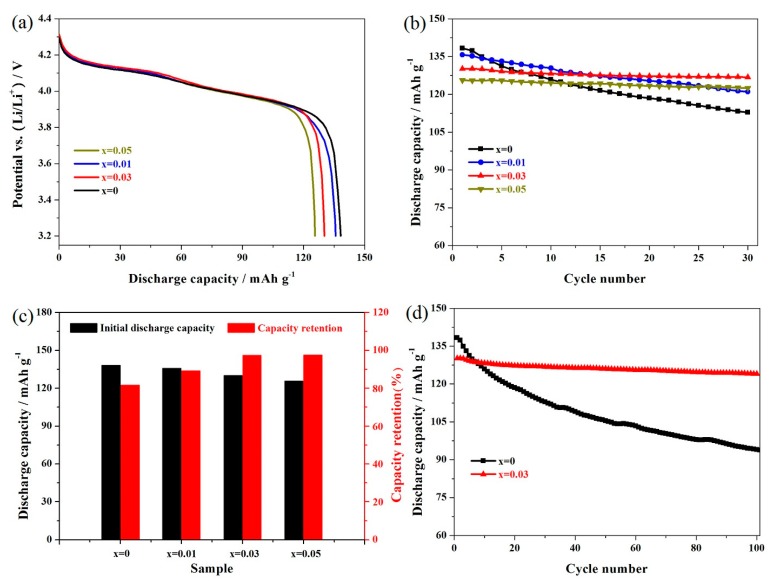
(**a**) Initial discharge curves and (**b**) cycling performance of the LiMn_2−x_Er_x_O_4_ (x = 0, 0.01, 0.03, 0.05) samples; (**c**) comparison plots of the initial discharge capacities and capacity retentions; and (**d**) long cycling performance of the LiMn_2−x_Er_x_O_4_ (x = 0, 0.03) samples.

Figure 6a presents the first discharge curves of these samples, which were tested at 0.5 C. All the Er-doped LiMn_2_O_4_ samples showed similar characteristic discharge curves to that of the undoped spinel. There were two distinct voltage platforms around 4.15 V and 4.00 V, suggesting that the introduction of the erbium ions did not change the electrochemical redox reaction mechanism, as all the LiMn_2−x_Er_x_O_4_ samples had two extraction/insertion steps of Li^+^ ions [29,43]. Figure 6b presents the cycling life of the LiMn_2−x_Er_x_O_4_ samples. The cycling life of the LiMn_2−x_Er_x_O_4_ (x = 0.01, 0.03, 0.05) samples was significantly improved as the erbium-doping amount increased because of the inhibition of the Jahn-Teller distortion and the improvement of the structural stability. Note, however, that the introduction of more erbium ions had a harmful effect on the reversible capacity of the LiMn_1.95_Er_0.05_O_4_ sample because of the reduction of the trivalent manganese ions. Figure 6c shows the comparison plots of the initial discharge capacities and capacity retentions of these samples. We can clearly observe the positive influence on the capacity retention and the adverse effect on the discharge capacity. These results indicate that introducing an appropriate amount of erbium ions can play an active role in enhancing the cycling life of a sample. Figure 6d presents the long cycling life of the undoped LiMn_2_O_4_ and LiMn_1.97_Er_0.03_O_4_ samples. For the optimal LiMn_1.97_Er_0.03_O_4_ sample, the initial reversible capacity could exhibit 130.2 mAh g^−1^. After 100 cycles, this sample exhibited 123.9 mAh g^−1^ with an outstanding retention of 95.2%. However, the undoped LiMn_2_O_4_ sample showed a poor cycling life with low reversible capacity after the 100th cycle. In particular, the undoped LiMn_2_O_4_ sample only delivered 93.7 mAh g^−1^ with a lower retention of 67.8% after 100 cycles. In addition, we compared the cycling performance of the LiMn_1.97_Er_0.03_O_4_ sample with that of the other doped samples, as shown in Table 2. It can be found that the erbium-doped LiMn_2_O_4_ sample show good cycling performance. These analyses further confirm the improvement of the cyclic stability by introducing some appropriate erbium ions into the spinel structure.

**Figure 7:**
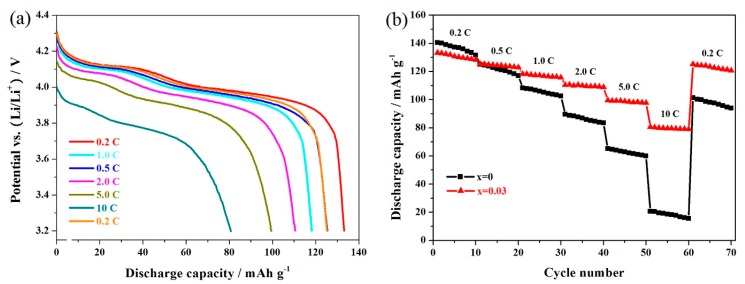
(**a**) Representative discharge curves of the LiMn_1.97_Er_0.03_O_4_ samples and (**b**) cycling performance of the LiMn_2−x_Er_x_O_4_ (x = 0, 0.03) samples at varying rates.

For the practical application of LiMn_2_O_4_, the rate performance is an important factor. The undoped LiMn_2_O_4_ and Er-doped LiMn_1.97_Er_0.03_O_4_ samples were tested successively at different rates. Figure 7a shows the corresponding discharge curves of the LiMn_1.97_Er_0.03_O_4_ samples. It can be seen that there were two voltage platforms, which were obvious at 0.2 C (the red color) and 0.5 C, suggesting the diffusion process of the lithium ions [20,33]. When the rate was further increased, these two potential plateaus gradually showed ambiguous boundaries and shifted toward the lower voltage when the cycling rate increased. This result has a lot to do with the polarization effect and ohmic drop [45,54]. Furthermore, when the cycling rate recovered to 0.2 C (the saffron yellow color), it was found that the LiMn_1.97_Er_0.03_O_4_ sample could show similar discharge capacity compared with the initial discharge capacity at 0.2 C (the red color), suggesting the excellent restorative performance of the LiMn_1.97_Er_0.03_O_4_ sample. Figure 7b shows the cycling stability of the undoped LiMn_2_O_4_ and the optimal LiMn_1.97_Er_0.03_O_4_ samples at varying rates. When cycled at 0.2 C, the capacities of these two samples reached up to 140.5 and 133.2 mAh g^−1^, respectively. However, what is important to pay attention to is the reversible capacity of the Er-doped LiMn_2_O_4_ sample. With the increasing of the cycling rate, these two samples can show much more different results. In particular, when cycled at 10 C, the LiMn_1.97_Er_0.03_O_4_ showed 80.7 mAh g^−1^, while the LiMn_2_O_4_ samples only showed 20.7 mAh g^−1^.

**Figure 8:**
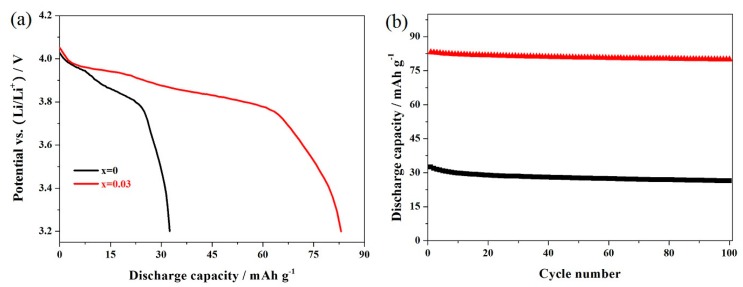
(**a**) Initial discharge curves and (**b**) cycling performance of the LiMn_2−x_Er_x_O_4_ (x = 0, 0.03) samples at 10 C.

Figure 8 shows the cycling performance of the LiMn_2_O_4_ and LiMn_1.97_Er_0.03_O_4_ samples at 10 C. As shown in Figure 8a, the high rate shows a greater negative impact on the characteristic voltage plateaus at around 4.15 and 4.0 V, respectively. For the LiMn_1.97_Er_0.03_O_4_ sample, these two voltage plateaus become blurred to a certain extent. What is worse, the LiMn_2_O_4_ sample presented a lower voltage plateau, and the capacity of the LiMn_2_O_4_ sample showed severe degradation. Figure 8b presents the cycling life of these two samples at 10 C. It can be found that the initial discharge capacity of the undoped LiMn_2_O_4_ sample only reached to 32.5 mAh g^−1^ with a poor capacity retention of 81.5%. By contrast, the optimal LiMn_1.97_Er_0.03_O_4_ sample displayed a higher discharge capacity of 83.1 mAh g^−1^. The discharge capacity still showed 78.0 mAh g^−1^ with an excellent capacity retention of 93.9%. These results suggest that the high-rate performance of LiMn_2_O_4_ can be enhance by doping manganese ions with erbium ions in the spinel structure.

**Figure 9:**
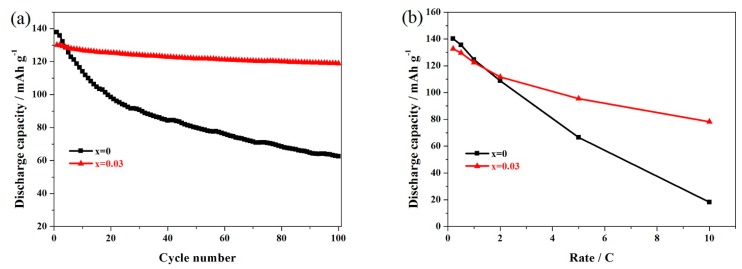
(**a**) Cycling performance and (**b**) rate capacities of the LiMn_2−x_Er_x_O_4_ (x = 0, 0.03) samples at 55 °C.

Figure 9a presents the cycling stability of the undoped LiMn_2_O_4_ and LiMn_1.97_Er_0.03_O_4_ samples at 55 °C. It can be seen from Figure 7a that the initial capacity of the LiMn_1.97_Er_0.03_O_4_ sample could reach up to 130.1 mAh g^−1^ at 0.5 C. Moreover, this sample still maintained a high capacity of 118.9 mAh g^−1^ with an excellent retention of 91.4% after 100 cycles. Unfortunately, the undoped LiMn_2_O_4_ sample showed very poor high-temperature cycling performance. After 100 cycles, the undoped sample only showed a lower capacity of 62.5 mAh g^−1^ with a low-capacity retention of 45.3%. These results suggest that introducing erbium ions can be favorable for enhancing the high-temperature performance of such a sample. Figure 9b shows the rate capability of these two samples at 55 °C. As shown here, the undoped LiMn_2_O_4_ and LiMn_1.97_Er_0.03_O_4_ samples showed similar capacities at low rates. However, these two samples presented obvious differences with the increasing of the rates. When cycled at 10 C, the LiMn_1.97_Er_0.03_O_4_ sample could exhibit 78.2 mAh g^−1^, while the LiMn_2_O_4_ sample only showed 18.3 mAh g^−1^. Based on these results, it can be concluded that the introduction of erbium ions can improve the high-temperature rate performance of LiMn_2_O_4_.

**Figure 10:**
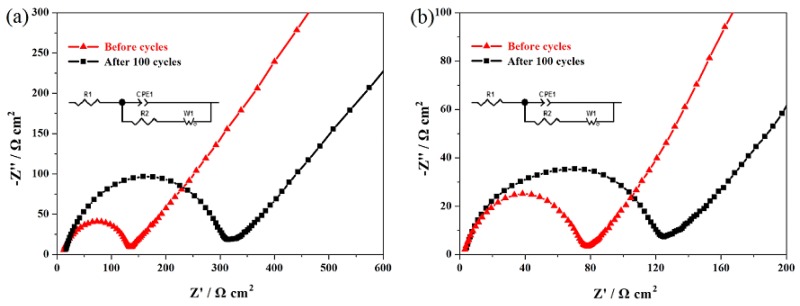
Nyquist plots of the LiMn_2_O_4_ (**a**) and LiMn_1.97_Er_0.03_O_4_ (**b**) samples before cycling and after 100 cycles.

Figure 10a,b show the EIS results of the undoped LiMn_2_O_4_ and LiMn_1.97_Er_0.03_O_4_ samples. As shown here, the high-frequency semicircle represents the charge transfer resistance (R_2_), which is closely related to the cycling life [21,48]. Thus, the effect of doping manganese ions with erbium ions on the cycling stability was mainly studied. The fitting values of R_2_ are listed in Table 3. For the LiMn_1.97_Er_0.03_O_4_ sample, the original R_2_ value only reached 73.4 Ω cm^2^ but increased to 115.1 Ω cm^2^ after 100 cycles. The R_2_ value increase was relatively small with a low growth rate of 56.8%. However, the undoped sample only showed the unsatisfactory R_2_ value. It can be seen that the undoped spinel showed a higher original R_2_ value (118.3 Ω cm^2^). After 100 cycles, the high growth rate reached up to 149.5% with the 100th R_2_ value of 295.2 Ω cm^2^. These results indicate that the addition of erbium ions in the spinel structure can have a positive role in decreasing the R_2_ value and enhancing the diffusion of lithium ions, which is conducive to the improvement of cycling stability [29,32].

## 4. Conclusions

In summary, we have successfully used the sol-gel technology to prepare the Er-doped LiMn_2_O_4_ samples. All these samples maintained the spinel structure of LiMn_2_O_4_ and showed relatively even particle size distribution. The optimal LiMn_1.97_Er_0.03_O_4_ sample showed a better cycling performance. When tested at 0.5 C, this sample delivered a reversible capacity of 130.2 mAh g^−1^ with an excellent retention of 95.2% after 100 cycles. At higher rate of 10 C, the reversible capacity of the LiMn_1.97_Er_0.03_O_4_ sample peaked at 83.1 mAh g^−1^, which is far higher than that of the undoped spinel. Moreover, this sample showed outstanding cycling stability at higher temperatures. All of these results indicate that the introduction of erbium ions could enhance the cycling stability of LiMn_2_O_4_.

## Figures and Tables

**Table 1:** Crystal parameters calculated from the XRD patterns of the LiMn_2−x_Er_x_O_4_ samples.

Sample	Space	a (nm)	Volume (nm^3^)	*I*_311_/*I*_400_
LiMn_2_O_4_	F*d*-3*m*	0.82334	0.55813	0.9054
LiMn_1.99_Er_0.01_O_4_	F*d*-3*m*	0.82291	0.55725	0.9513
LiMn_1.97_Er_0.03_O_4_	F*d*-3*m*	0.82177	0.55494	0.9917
LiMn_1.95_Er_0.05_O_4_	F*d*-3*m*	0.82053	0.55244	0.9983

**Table 2:** Comparison of the various doped-LiMn_2_O_4_ samples described in the references including this work.

Sample	Synthesis Method	Initial Capacity and Capacity Retention	Ref.
**Li(Li_0.06_Mn_1.94_)O_4_**	Solid-state method	123.0 mAh g^−1^, 86.6% after 200 cycles at 0.5 C (25 °C)	[34]
**Li(Zn_0.05_Mn_1.95_)O_4_**	Solution combustion method	102.6 mAh g^−1^, 82.9% after 500 cycles at 1.0 C (25 °C)	[20]
**Li(Mg_0.08_Mn_1.92_)O_4_**	Solid-state combustion synthesis	101.3 mAh g^−1^, 98.1% after 40 cycles at 0.2 C (ambient temperature)	[43]
**Li(Cu_0.05_Mn_1.95_)O_4_**	Molten-salt combustion method	119.0 mAh g^−1^, 95.0% after 100 cycles at 0.5 C	[31]
**Li(Al_0.06_Mn_1.94_)O_4_**	Co-precipitation method	117.4 mAh g^−1^, 97.0% after 100 cycles at 1.0 C (55 °C)	[44]
**Li(Cr_0.05_Mn_1.95_)O_4_**	Citric acid-assisted combustion method	117.0 mAh g^−1^, 81.2% after 100 cycles at 0.5 C	[55]
**Li(Co_0.02_Mn_1.97_)O_4_**	Controlled crystallization method	116.8 mAh g^−1^, 91.0% after 350 cycles at 296 mA g^−1^	[56]
**Li(Ti_0.03_Mn_1.97_)O_4_**	Solid-state method	135.7 mAh g^−1^, 95.0% after 70 cycles at 0.5 C (room temperature)	[38]
**Li(Si_0.05_Mn_1.97_)O_4_**	Solid-state method	134.6 mAh g^−1^, 85.1% after 100 cycles at 0.5 C (room temperature)	[29]
**Li(Zr_0.02_Mn_1.97_)O_4_**	Solid-state method	113.8 mAh g^−1^, 95.5% after 50 cycles at 0.2 C (25 °C)	[57]
**Li(Er_0.03_Mn_1.97_)O_4_**	Sol-gel method	130.2 mAh g^−1^, 95.2% after 100 cycles at 0.5 C (room temperature)	This work

**Table 3:** Fitting values of the charge transfer resistance (R_2_) calculated from EIS.

Sample	R_2_ (Ω cm^2^) before Cycles	R_2_ (Ω cm^2^) after 100 Cycles	Percentage of Increase
LiMn_2_O_4_	118.3	295.2	149.5%
LiMn_1.97_Er_0.03_O_4_	73.4	115.1	56.8%

## References

[1] Park O.K., Cho Y., Lee S., Yoo H.-C., Song H.-K., Cho J. (2011). Who will drive electric vehicles, olivine or spinel?. Energy Environ. Sci..

[2] Zhao H., Wang J., Wang G., Liu S., Tan M., Liu X., Komarneni S. (2017). Facile synthesis of orthorhombic LiMnO_2_ nanorods by in-situ carbothermal reduction: Promising cathode material for Li ion batteries. Ceram. Int..

[3] Blomgren G.E. (2016). The development and future of lithium ion batteries. J. Electrochem. Soc..

[4] Normakhmedov O.O., Brylev O.A., Petukhov D.I., Kurilenko K.A., Kulova T.L., Tuseeva E.K., Skundin A.M. (2018). Cryochemically processed Li_1+y_Mn_1.95_Ni_0.025_Co_0.025_O_4_ (y = 0, 0.1) cathode materials for Li-ion batteries. Materials.

[5] Scrosati B., Garche J. (2010). Lithium batteries: Status, prospects and future. J. Power Sources.

[6] Zhao H., Liu S., Liu X., Tan M., Wang Z., Cai Y., Komarneni S. (2016). Orthorhombic LiMnO_2_ nanorods as cathode materials for lithium-ion batteries: Synthesis and electrochemical properties. Ceram. Int..

[7] Han C.G., Zhu C., Saito G., Sheng N., Nomura T., Akiyama T. (2017). Enhanced cycling performance of surface-doped LiMn_2_O_4_ modified by a Li_2_CuO_2_-Li_2_NiO_2_ solid solution for rechargeable lithium-ion batteries. Electrochim. Acta.

[8] Bakierska M., Świętosławski M., Gajewska M., Kowalczyk A., Piwowarska Z., Chmielarz L., Dziembaj R., Molenda M. (2016). Enhancement of electrochemical performance of LiMn_2_O_4_ spinel cathode material by synergetic Substitution with Ni and S. Materials.

[9] Quinlan R.A., Lu Y.C., Kwabi D., Shao-Horn Y., Mansour A.N. (2015). XPS Investigation of the electrolyte induced stabilization of LiCoO_2_ and “AlPO_4_”-coated LiCoO_2_ composite electrodes. J. Electrochem. Soc..

[10] Xiao X., Liu X., Wang L., Zhao H., Hu Z., He X., Li Y. (2012). LiCoO_2_ nanoplates with exposed (001) planes and high rate capability for lithium-ion batteries. Nano Res..

[11] Amin R., Lin C., Peng J., Weichert K., Acartürk T., Starke U., Maier J. (2009). Silicon-doped LiFePO_4_ single crystals: growth, conductivity behavior, and diffusivity. Adv. Funct. Mater..

[12] Oh S.W., Myung S.T., Oh S.M., Oh K.H., Amine K., Scrosati B., Sun Y.K. (2010). Double carbon coating of LiFePO_4_ as high rate electrode for rechargeable lithium batteries. Adv. Mater..

[13] Cook J.B., Kim C., Xu L., Cabana J. (2013). The effect of Al substitution on the chemical and electrochemical phase stability of orthorhombic LiMnO_2_. J. Electrochem. Soc..

[14] He Y., Feng Q., Zhang S., Zou Q., Wu X., Yang X. (2013). Strategy for lowering Li source dosage while keeping high reactivity in solvothermal synthesis of LiMnO_2_ nanocrystals. ACS Sustain. Chem. Eng..

[15] Zhao H., Li D., Wang Y., Li F., Wang G., Wu T., Wang Z., Li Y., Su J. (2018). Sol-gel synthesis of silicon-doped lithium manganese oxide with enhanced reversible capacity and cycling stability. Materials.

[16] Lu J., Zhou C., Liu Z., Lee K.S., Lu L. (2016). LiMn_2_O_4_ cathode materials with large porous structure and radial interior channels for lithium ion batteries. Electrochim. Acta.

[17] Chen M., Chen P., Yang F., Song H., Liao S. (2016). Ni, Mo co-doped lithium manganate with significantly enhanced discharge capacity and cycling stability. Electrochim. Acta.

[18] Gao X., Sha Y., Lin Q., Cai R., Tade M.O., Shao Z. (2015). Combustion-derived nanocrystalline LiMn_2_O_4_ as a promising cathode material for lithium-ion batteries. J. Power Sources.

[19] Hao J., Bai H., Liu J., Yang F., Li Q., Su C., Guo J. (2016). Synthesis and electrochemical properties of spinel Li(Li_0.05_Cu_0.05_Mn_1.90_)O_4_ by a flameless combustion method. J. Alloys Compd..

[20] Xu W., Li Q., Guo J., Bai H., Su C.W., Ruan R., Peng J. (2016). Electrochemical evaluation of LiZn_x_Mn_2−x_O_4_ (x ≤ 0.10) cathode material synthesized by solution combustion method. Ceram. Int..

[21] Zhao H., Liu S., Wang Z., Cai Y., Tan M., Liu X. (2016). Enhanced elevated-temperature performance of LiAl_x_Si_0.05_Mg_0.05_Mn_1.90−x_O_4_ (0 ≤ x ≤ 0.08) cathode materials for high-performance lithium-ion batteries. Electrochim. Acta.

[22] Capsoni D., Bini M., Chiodelli G., Mustarelli P., Massarotti V., Azzoni C.B., Mozzati M.C., Linati L. (2002). Inhibition of Jahn-Teller cooperative distortion in LiMn_2_O_4_ spinel by Ga^3+^ doping. J. Phys. Chem. B.

[23] Han C.G., Zhu C., Saito G., Akiyama T. (2016). Improved electrochemical performance of LiMn_2_O_4_ surface-modified by a Mn^4+^-rich phase for rechargeable lithium-ion batteries. Electrochim. Acta.

[24] Zhao H., Li F., Bai X., Wu T., Wang Z., Li Y., Su J. (2018). Enhanced Cycling Stability of LiCu_x_Mn_1.95−x_Si_0.05_O_4_Cathode Material Obtained by Solid-State Method. Materials.

[25] Shang Y., Lin X., Lu X., Huang T., Yu A. (2015). Nano-TiO_2_(B) coated LiMn_2_O_4_ as cathode materials for lithium-ion batteries at elevated temperatures. Electrochim. Acta.

[26] Zhang C., Liu X., Su Q., Wu J., Huang T., Yu A. (2017). Enhancing electrochemical performance of LiMn_2_O_4_ cathode material at elevated temperature by uniform nanosized TiO_2_ coating. ACS Sustain. Chem. Eng..

[27] Peng Z., Wang G., Cao Y., Zhang Z., Du K., Hu G. (2016). Enhanced high power and long life performance of spinel LiMn_2_O_4_ with Li_2_MnO_3_ coating for lithium-ion batteries. J. Solid State Electrochem..

[28] Zhao H., Li F., Liu X., Cheng C., Zhang Z., Wu Y., Xiong W., Chen B. (2015). Effects of equimolar Mg (II) and Si (IV) co-doping on the electrochemical properties of spinel LiMn_2−2x_Mg_x_Si_x_O_4_ prepared by citric acid assisted sol-gel method. Electrochim. Acta.

[29] Zhao H., Liu S., Wang Z., Cai Y., Tan M., Liu X. (2016). LiSi_x_Mn_2−x_O_4_ (x ≤ 0.10) cathode materials with improved electrochemical properties prepared via a simple solid-state method for high-performance lithium-ion batteries. Ceram. Int..

[30] Feng X., Zhang J., Yin L. (2016). Effect of AlP coating on electrochemical properties of LiMn_2_O_4_ cathode material for lithium ion battery. Mater. Res. Bull..

[31] Huang J.J., Li Q.L., Bai H.L., Xu W., He Y., Su C., Peng J.H., Guo J. (2015). Preparation and electrochemical properties of LiCu_x_Mn_2−x_O_4_ (x ≤ 0.10) cathode material by a low temperature molten-salt combustion method. Int. J. Electrochem. Sci..

[32] Zhang H., Xu Y., Liu D., Zhang X., Zhao C. (2014). Structure and performance of dual-doped LiMn_2_O_4_ cathode materials prepared via microwave synthesis method. Electrochim. Acta.

[33] Zhao H., Liu S., Cai Y., Wang Z., Tan M., Liu X. (2016). A simple and mass production preferred solid-state procedure to prepare the LiSi_x_Mg_x_Mn_2−2x_O_4_ (0 ≤ x ≤ 0.10) with enhanced cycling stability and rate capability. J. Alloys Compd..

[34] Yu F.D., Wang Z.B., Chen F., Wu J., Zhang X.G., Gu D.M. (2014). Crystal structure and multicomponent effects in Li_1+x_Mn_2−x−y_Al_y_O_4_ cathode materials for Li-ion batteries. J. Power Sources.

[35] Guo D., Li B., Chang Z., Tang H., Xu X., Chang K., Shangguan E., Yuan X.Z., Wang H. (2014). Facile synthesis of LiAl_0.1_Mn_1.9_O_4_ as cathode material for lithium ion batteries: Towards rate and cycling capabilities at an elevated temperature. Electrochim. Acta.

[36] Mohan P., Ranjith B., Kalaignan G.P. (2014). Structure and electrochemical performances of co-substituted LiSm_x_La_0.2−x_Mn_1.80_O_4_ cathode materials for rechargeable lithium-ion batteries. J. Solid State Electrochem..

[37] Jayapal S., Mariappan R., Sundar S., Piraman S. (2014). Electrochemical behavior of LiMn_2−X−Y_Ti_X_Fe_Y_O_4_ as cathode material for Lithium ion batteries. J. Electroanal. Chem..

[38] Xiong L., Xu Y., Zhang C., Zhang Z., Li J. (2010). Electrochemical properties of tetravalent Ti-doped spinel LiMn_2_O_4_. J. Solid State Electrochem..

[39] Zhan D., Yang F., Zhang Q., Hu X., Peng T. (2014). Effect of solid-state reaction temperature on electrochemical performance of LiMn_2_O_4_ submicro-rods as cathode material for Li-ion battery by using γ-MnOOH submicro-rods as self-template. Electrochim. Acta.

[40] Zou H., Wang B., Wen F., Chen L. (2017). Hydrothermal synthesis of pure LiMn_2_O_4_ from nanostructured MnO_2_ precursors for aqueous hybrid supercapacitors. Ionics.

[41] Chen K., Donahoe A.C., Noh Y.D., Li K., Komarneni S., Xue D. (2014). Conventional- and microwave-hydrothermal synthesis of LiMn_2_O_4_: Effect of synthesis on electrochemical energy storage performances. Ceram. Int..

[42] Huang J., Yang F., Guo Y., Peng C., Bai H., Peng J., Guo J. (2015). LiMg_x_Mn_2−x_O_4_ (x ≤ 0.10) cathode materials with high rate performance prepared by molten-salt combustion at low temperature. Ceram. Int..

[43] Xiang M., Ye L., Peng C., Zhong L., Bai H., Su C., Guo J. (2014). Study on the electrochemical performance of high-cycle LiMg_0.08_Mn_1.92_O_4_ cathode material prepared by a solid-state combustion synthesis. Ceram. Int..

[44] Yi X., Wang X., Ju B., Wei Q., Yang X., Zou G., Shu H., Hu L. (2014). Elevated temperature cyclic performance of LiAl_x_Mn_2−x_O_4_ microspheres synthesized via co-precipitation route. J. Alloys Compd..

[45] Zhao H., Liu X., Cheng C., Li Q., Zhang Z., Wu Y., Chen B., Xiong W. (2015). Synthesis and electrochemical characterizations of spinel LiMn_1.94_MO_4_ (M = Mn_0.06_, Mg_0.06_, Si_0.06_, (Mg_0.03_Si_0.03_)) compounds as cathode materials for lithium-ion batteries. J. Power Sources.

[46] Thirunakaran R., Lew G.H., Yoon W.S. (2016). Cerotic acid assisted sol-gel synthesis and electrochemical performance of double doped spinels (LiCr_x_Mg_y_Mn_2−x−y_O_4_) as cathode materials for lithium rechargeable batteries. Powder Technol..

[47] Wang Z., Du J., Li Z., Wu Z. (2014). Sol-gel synthesis of Co-doped LiMn_2_O_4_ with improved high-rate properties for high-temperature lithium batteries. Ceram. Int..

[48] Xiong L., Xu Y., Tao T., Goodenough J.B. (2012). Synthesis and electrochemical characterization of multi-cations doped spinel LiMn_2_O_4_ used for lithium ion batteries. J. Power Sources.

[49] Zhang H., Liu D., Zhang X., Zhao C., Xu Y. (2013). Microwave synthesis of LiMg_0.05_Mn_1.95_O_4_ and electrochemical performance at elevated temperature for lithium-ion batteries. J. Solid State Electrochem..

[50] Liu H., Song L., Zhang K. (2005). Er-Doped LiMn_2_O_4_. Inorg. Mater..

[51] Zhao H., Li F., Liu X., Xiong W., Chen B., Shao H., Que D., Zhang Z., Wu Y. (2015). A simple, low-cost and eco-friendly approach to synthesize single-crystalline LiMn_2_O_4_ nanorods with high electrochemical performance for lithium-ion batteries. Electrochim. Acta.

[52] Wang J.L., Li Z.H., Yang J., Tang J.J., Yu J.J., Nie W.B., Lei G.T., Xiao Q.Z. (2012). Effect of Al-doping on the electrochemical properties of a three-dimensionally porous lithium manganese oxide for lithium-ion batteries. Electrochim. Acta.

[53] Sun Y., Zhao Z., Li P., Li G., Chen Y., Zhang W., Hu J. (2015). Er-doped ZnO nanofibers for high sensibility detection of ethanol. Appl. Surf. Sci..

[54] Ding Y.L., Xie J., Cao G.S., Zhu T.J., Yu H.M., Zhao X.B. (2011). Single-crystalline LiMn_2_O_4_ nanotubes synthesized via template-engaged reaction as cathodes for high-power lithium ion batteries. Adv. Funct. Mater..

[55] Du K., Xie J., Wang J., Zhang H. (2003). LiMn_2−x_Cr_x_O_4_ spinel prepared by a modified citrate route with combustion. J. Power Sources.

[56] Jiang J., Du K., Cao Y., Peng Z., Hu G. (2015). Synthesis of the micro-spherical LiMn_2−x_Co_x_O_4_ as cathode material of lithium batteries. J. Nanosci. Nanotechnol..

[57] Tang Z.Y., Zhang N., Lu X.H., Huang Q.H. (2005). Characterizations of spinel LiMn_2−x_Zr_x_O_4 C_athode for lithium-ion batteries. Acta Phys.-Chim. Sin..

